# Direct Probing of Dispersion Quality of ZrO_2_ Nanoparticles Coated by Polyelectrolyte at Different Concentrated Suspensions

**DOI:** 10.1186/s11671-015-1157-z

**Published:** 2015-12-01

**Authors:** Hamid Sarraf, Zhenghua Qian, Ludmila Škarpová, Bin Wang, Reinhard Herbig, Martin Maryška, Lidmila Bartovska, Jiří Havrda, Bahman Anvari

**Affiliations:** State Key Laboratory of Mechanics and Control of Mechanical Structures, Nanjing University of Aeronautics and Astronautics, 29 Yudao Street, Nanjing, 210016 China; Western Governors University, 1001 Fourth Avenue, Seattle, WA 98154-1101 USA; NANOPRODEX LLC, NW, 48th Ave, Vancouver, WA 98685 USA; University of Chemistry and Technology, 166 28 Prague, Czech Republic; Institute of Electronic and Sensor Materials, TU Bergakademie Freiberg, Gustav-Zeeunter-Str. 3, Freiberg, 09596 Germany; Department of Bioengineering, 900 University Ave., University of California, Riverside, CA 92521 USA

**Keywords:** ZrO_2_ nanoparticle, Colloidal dispersion, Anionic polyelectrolyte dispersant DOLAPIX CE64, Viscosity, Electrokinetic sonic amplitude (ESA) technique, Zeta-potential, SEM, TEM, 81. Materials science, 83. Rheology, 43. Acoustics

## Abstract

This study reports useful application of the electrokinetic sonic amplitude (ESA) technique in combination with rheometry and electron microscopy techniques for direct probing the stability of low and high-concentrated zirconia (ZrO_2_) nanosuspensions in the presence of an alkali-free anionic polyelectrolyte dispersant Dolapix CE64. A comparative study of the electrokinetic characteristics and the rheological behavior of concentrated ZrO_2_ nanosuspensions has been done. Good agreement was obtained from relationship between the electrokinetic characteristics (zeta potential, ESA signal), viscosity, and its pH dependence for each concentrated ZrO_2_ nanosuspension with different dispersant concentration in the range of 0.9–1.5 mass%. A nanoscale colloidal hypothesis is proposed to illustrate that the addition of different amounts of dispersant influences on both the stability and the electrokinetic and rheological properties of concentrated ZrO_2_ nanosuspensions. It is found that an optimum amount of 1.4 mass% dispersant at the inherent pH (>9.2) can be attached fully onto the nanoparticles with sufficient electrosteric dispersion effects, suitable for casting applications. Supplementary scanning electron microscopy (SEM) and high-resolution transmission electron microscopy (HR-TEM) analyses followed by colorization effect were taken to verify the visible interaction between dispersant and nanoparticles surfaces. SEM and HR-TEM images proved the existence of visible coverage of dispersant on the surface of individual nanoparticles and showed that thin polyelectrolyte layers were physically bound onto the particles’ surfaces. This study will be of interest to materials scientists and engineers who are dealing with dispersion technology, nanoparticle surface treatments, functionalization, characterization, and application of bio/nanoparticle suspensions at various concentrations using different types of polymers.

## Background

Dispersion of nanoparticles in liquids and characterization of their dispersion stabilities at different colloidal concentrations are not only on the center stage in ceramic processing methods, such as tape casting [1], slip casting [2–4], and direct ink writing [5], but also in nano/biotechnology development [6–8]. Ceramic colloidal processing methods require stable, well-dispersed suspensions with extremely high levels of solids loading to achieve optimal casting condition and green body properties and also to minimize drying induced shrinkage [9–11]. Several techniques are available to characterize the dispersion quality of *micron* and *sub-micron*-sized powder suspensions at low solids loading. These include rheological, sedimentation, adsorption, electrophoresis, charge quantity, and zeta (ζ)-potential measurements or optical light scattering techniques [4, 12, 13]. However, there are no many standard techniques available for dispersion characterization of nanoparticles suspensions at high (>2 vol.%) solids loading. One of the available techniques that can be used for characterization of the electrokinetic properties of dispersed nanoparticle suspensions is laser light scattering technique. But, the majority of standard laser light scattering techniques are *indirect.*

In addition, applying existing light scattering techniques has several limitations: (i) The light scattering techniques require dilution of the concentrated suspension. This may lead to the shift of the equilibria involving adsorption and may be irreversible, unless particular care is taken to compensate for the dilution process [13]. (ii) The light scattering techniques are limited only for characterization of the electrokinetic properties of very low-concentrated (i.e., solids loading <1 vol.%) suspensions of solids [12, 14, 15]. Because of recent advances in purity, high-strength, high-fracture toughness and wear resistance over a wide temperature range, and other versatile applications, zirconia (ZrO_2_) has become an important ceramic material [7, 16] for a variety of applications. It is being used as an important material for Orthopedic and dental applications [17], electrolytes and electrochemical sensors [18, 19], gas and humidity sensors [20, 21], anode in fuel cells [22], catalysts [23], and optoelectrics [24]. Thus, due to the above important characteristics of zirconia, in this study, we used high-purity ZrO_2_ nanopowder.

For fabrication of ZrO_2_-based ceramic materials with final sintered properties of high tetragonal-phase (*t*-phase) content, along with dense and uniform microstructure leading to good mechanical properties, it is preferable to start with a concentrated nanopowder suspension with an appropriate dispersant stabilizer [25]. Some of the dispersants which have been used to disperse sub-micron-sized zirconia powder include Tiron, Aluminon [26], ammonium polyacrylate [27], polymethacrylic acid, polyethyleneimine and diammonium citrate [28], 2-propenoic acid and a metal-organic polymer [29], and Triton-X114 [30]. Greenwood and Kendall [31] have conducted a research study using 14 different reagents for dispersing sub-micron-sized zirconia powder. Although several dispersants have been applied for dispersion of low-concentrated suspensions, direct probing of the effect of dispersant concentration and its subsequent effect on the rheological and electrokinetic properties of higher-concentrated (>70 mass% or 30 vol.%) suspensions of ZrO_2_ nanoparticles are hitherto unreported, specifically by using advanced electroacoustic technique.

Electroacoustic technique is extremely influential in determining nanoparticle suspension stability by measuring the magnitude of the nanoparticle charge, known as ζ-potential, at different pH levels. If the magnitude of the ζ-potential to the isoelectric point (IEP) is high enough, then the net particle-particle interaction will be repulsive and can prevent particles from agglomerating [12, 32]. A higher magnitude of ζ-potential usually leads to stable suspensions with lower viscosity. Lower viscosity will lead to a higher packing density in the sediment [32, 33]. The zeta-potential can be modified by adjusting the suspension pH and by using suitable dispersants, such as polyelectrolytes. However, conventional polyelectrolytes like polyacrylic acid (PAA), polymethacrylic acid (PMAA), or sodium dodecyl sulfate (SDS) are weak dispersants based on steric or electrostatic stabilization and not suitable for dispersion of high-concentrated nanoparticle suspensions [34–36]. In addition, solids loading and using suitable dispersant have also important impact on dispersion quality of concentrated colloidal nanoparticle suspensions. Several studies have been reported about the effects of volume fraction and the adsorption of different conventional polyelectrolytes onto different sub-micron-sized powders like calcium pyrophosphate [34], alumina, kaolin [37–39], and barium titanate (BaTiO_3_) at different low and moderate-concentrated suspensions [9, 35].

The desirable combination is to apply a dispersant which can adsorb strongly onto the ceramic nanoparticles at high solids loading suspensions with optimal viscosity. Therefore, one of the most important concerns in this study was to use the most suitable dispersant that could stabilize highly concentrated suspensions of ZrO_2_ nanoparticles based on electrosteric stabilization with ultimate goal for fabrication of dense zirconia ceramic with homogenous microstructure and improved mechanical properties. Recently, we have reported characterization of both rheological and electrokinetic properties of high-concentrated α-Al_2_O_3_ [4, 12] and Al_2_O_3_-ZrO_2_ [40] ceramic suspensions by using a new type of anionic dispersant Dolapix CE64 (Zschimmer & Schwarz GmbH Co., Germany) functioning on the principle of electrosteric stabilization. According to the results reported by manufacturer and different authors [4, 12, 26–32, 40–45], this particular dispersant has several advantages over traditional dispersants. Specifically, Dolapix CE64 has the following unique properties:

(I) Dolapix CE64 is an alkali-free anionic polyelectrolyte dispersant which does not foam. Specially, it has minimum side effect on gypsum-based casting molds used for shaping final products. Therefore, this dispersant makes it possible to produce slips with a high solids loading, particularly, suitable for dispersion before slip casting, tape casting, and spray drying.
(II) It is a liquid and its density is just slightly above the density of water. Comparing to other types of dispersant agents, it has lower molecular weight and higher pH of around 9 that helps better absorption coverage of nanoparticles in concentrated colloidal systems.
(III) Since Dolapix CE64 is liquid and is thus completely dissociated in water, the electrosteric stabilization effect commences immediately after addition to the suspension/slip. Hence, it is possible to any time to adjust the viscosity of the suspension by rapid, homogeneous incorporation into the suspension. Electrosteric stabilization offers the advantage of a double stabilization compared to electrostatic stabilization, indicating that not only the electrostatic interactions at the solid/liquid (S/L) interface are under control but also steric repulsion caused by the molecular architecture is considered [45, 46]. For this matter, dispersant Dolapix CE64 was used in this study as the most reliable candidate for this mission.

Very few researchers [43, 47] have reported dispersion of only up to 65 mass% solids loading of sub-micron ZrO_2_ suspensions using Dolapix CE64. Rao et al. [43] have carried out only conventional sedimentation and capillary suction time measurements to evaluate the effect of pH, concentration of polyelectrolyte and solids loading on the dispersion of sub-micron ZrO_2_ particles. However, these techniques are indirect methods and time consuming processes. E. Özkol et al. [47] have carried out viscosity measurements using rotational rheometer and a conventional laser-scattering method for particle size characterization of ZrO_2_ suspensions of only up to 65 mass% (24.2 vol.%) solids loading.

In this study, the possibility of direct probing dispersion quality and measuring the effect of anionic polyelectrolyte (Dolapix CE64) concentration (mass%) on the electrokinetic (ζ-potential, the relative ESA signal) and rheological (viscosity) properties with pH dependency of variously concentrated ZrO_2_ nanosuspensions was investigated. For this purpose, two series of low (10 mass%, ~2 vol.%)- and high (77 mass%, ~35.8 vol.%)-concentrated ZrO_2_ nanosuspensions were prepared. Powerful tools like Haake rheometer and electrokinetic sonic amplitude (ESA) device (Matec, Applied Science, USA) [44] were applied.

The results obtained from rheology were compared with those obtained using the electroacoustic ESA technique. Imaging and characterization of nanoparticles by high-resolution electron microscopy techniques are also important tools in multi-component material characterization [48]. Such techniques are used to assess nanoparticle shape, surface, size, morphology, coating, and elemental distributions [49]. Therefore, in this study, we also applied SEM and HR-TEM as supplementary tools to verify the visible interaction between polyelectrolyte dispersant and nanoparticle surfaces, qualitatively.

## Methods

### Powder Characterization

**Fig. 1:**
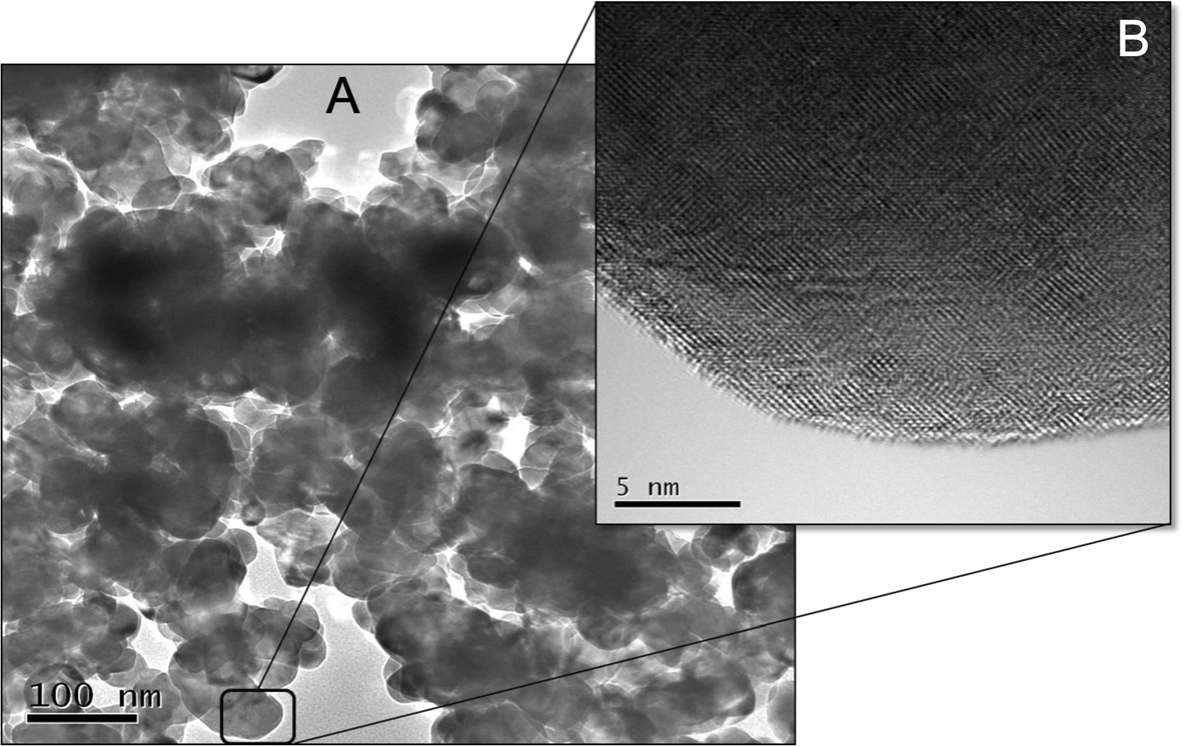
**a**, **b** A TEM image of aggregated bare ZrO_2_ nanoparticles (**a**) and an indexed HR-TEM image show bare surface and visible lattices of an bare ZrO_2_ nanoparticle (**b**)

ZrO_2_ nanopowder (99.99 % high purity, Tosoh Corp.) was used as starting material. Morphology of the powder with a primary crystallite size in the range of 25–60 nm (Fig. 1a) was verified by transmission electron microscope (FE-TEM, JEOL JEM-2010 F, Center for Analysis of Advanced Materials, Tokyo Institute of Technology). Nanopowder has a bulk density of 6.05 g/cm^3^ and surface area (by BET) of (13.0–19.0) 16.0 m^2^/g. A high-resolution TEM image of individual crystalline ZrO_2_ nanoparticle is shown in Fig. 1b.

### Dispersant Selection

**Fig. 2:**
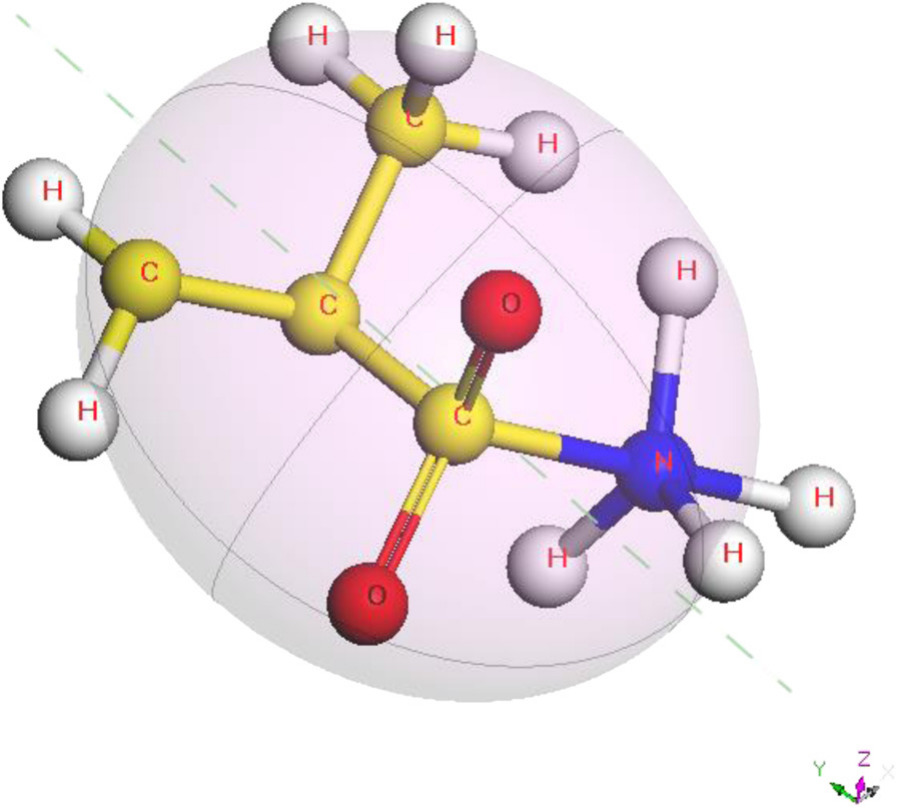
An orthographic display of predicted 3-D molecular structure of alkali-free anionic polymer, Dolapix CE64

A new type of electrosteric dispersant, Dolapix CE64 (Zschimmer & Schwarz GmbH Co., Germany), was used in this study as a dispersing agent. Table 1 introduces several physical characteristics of Dolapix CE64 that are provided by the manufacturer. The manufacturer does not give further information about this dispersant. A predicted orthographic display of the 3-D molecular structure (simulated by using materials studio, Accelrys) of this dispersant is illustrated in Fig. 2. In this study, dispersant concentration is expressed in mass% on dry powder basis. Solids loading is expressed in both mass% and volume%.

**Table 1:** Characteristics of dispersant Dolapix CE64 (as provided by manufacturer)

Appearance	Yellowish viscous solution
Active matter	Approximately 65 %
Solubility	Water-miscible
Density (20 °C)	Approximately 1.2 g/cm^3^
Active pH area (20 °C)	Approximately 9
Molecular weight	~660 g/mol
Optimal dosage formulation (mass%)	(−)
Nomenclature ionic groups	Synthetic polyelectrolyte; alkali-free

*(−)* product information not provided by manufacturer

### Suspension Preparation

To determine viscosity, ζ-potential and ESA signal as a function of pH, series of nanosuspensions were applied. Several samples (each of the volume of 120 ml) of ZrO_2_ nanopowder suspensions were prepared in distilled water in concentrations of 2 and 35.8 vol.% (10 and 77 mass%, respectively). One set of samples was prepared with and the others without addition of Dolapix CE64 in the range of between 0.9–1.5 mass%. The resulting nanosuspensions were treated in 250 ml zirconia milling jars with 100 g of spherical 3–8 mm zirconia ceramic balls as milling media. Samples were then mixed in a planetary ball mill (Pulverisette 6, Fritsch, Germany) for a period of 30 min with 500 rpm [12]. Subsequently, after milling, the resulting suspensions were cooled to room temperature (23 °C), and de-agglomeration was additionally performed in vacuum (laboratory desiccator) for 5 min to remove gas bubbles. Suspensions were ultrasonicated under an output power of 200 W for a period of 5 min and again degassed for an additional 5 min for better homogeneity, which is essential prior to rheological and electrokinetic (ESA) measurements, as well as for slip casting application. At least ten suspensions were prepared for each different nanosuspension composition, in order to determine the optimal amount of dispersant which would give the lowest viscosity and control reproducibility of suspensions. Immediately, fresh nanosuspensions were evaluated with respect to their rheology (by means of viscosity measurement), ζ-potential (by means of ESA and pH measurements), and particle morphology (by using SEM and HR-TEM microscopes).

### Suspension Characterization

#### Rheology Measurement

To stabilize high solids loading (in this work: *ϕ* = 77 mass%) ZrO_2_ nanosuspensions, it is necessary to achieve the optimum dispersant concentration. The effect of dispersant concentration in the range of 0.9–1.5 mass% on the rheological behaviors [i.e., flow curves: by means of shear stress, (τ)-vs-shear rate, (γ) and viscosity (*η*)] of high-concentrated ZrO_2_ nanosuspensions with constant 77 mass% solids loading (*ϕ*) was evaluated at different shear rates (γ) between 0.03–1000 (s^−1^). Experimentally, suspension with optimum dispersant concentration displays optimum viscosity at a shear rate of 50 s^−1^ with high stability, and it can be handled easily for colloidal processing techniques such as aqueous gel and slip casting of ceramics [4, 50, 51].

In addition to determining the optimal viscosity, the influence of pH and dispersant concentration on the viscosity of concentrated ZrO_2_ nanosuspensions (with constant 77 mass% solids loading) at shear rate of 50 s^−1^ was investigated, separately. Separate samples were prepared at the required dispersant concentrations (in the range of 0.9–1.5 mass%) and pH. Immediately, rheological measurements of all formulated suspensions were carried out at 23 °C by pouring suspensions into a concentric cylindrical rheometer (Thermo Haake Ltd., Sensor Z41-DIN measurement system, RV1, Germany). A volume of 14 ml was used in the cylinder for each measurement. To avoid undesired influence from different mechanical histories, the fresh samples were presheared at an identical rate of 100 s^−1^ for 1 min, followed by an equilibrium period of 2 min prior to viscosity measurement. Three measurements were made for each suspension, and each result was identical on the whole. Immediately, the viscosity of the suspensions was determined.

#### Principles of Zeta-Potential Measurement, Using “ESA” Technique

Like other electroacoustic techniques [52, 53], the electroacoustic sonic amplitude “ESA” technique is well suited for studying the colloidal behavior of advanced ceramic materials and the evaluation of the electrokinetic properties of concentrated nanoparticle systems [12, 32, 44, 54–56].

**Fig. 3:**
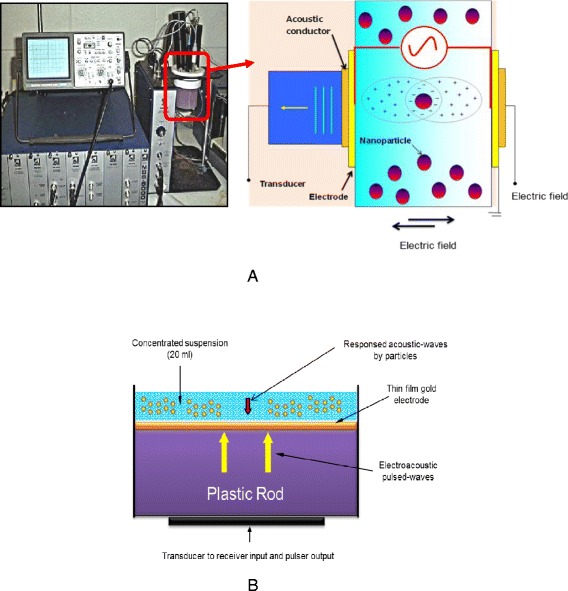
**a**, **b** Schematic illustration of ESA instrument and its measurement cell, showing polarization of electric double layer for negatively charged nanoparticles suspended in polar medium and subjected to an AC electric field

The principles of electroacoustic techniques and theoretical background of ESA technique and its application in concentrated colloidal suspensions have already been reported in details by several authors [56–58]. According to electroacoustic theory, when an alternating electric field is applied to a suspension of charged particles, the particles will oscillate at the same frequency of the applied field [59]. Due to the density difference between the particles and the aqueous suspension, this motion will generate tiny acoustic dipoles associated with each individual particle [12, 57, 58]. These dipoles cancel one another throughout the body of suspension except near the electrodes where the acoustic dipoles generate an acoustic wave. This can emerge from the suspension and move down the delay rod where it is detected by the transducer. The transducer can detect the amplitude and phase angle of this acoustic wave as a function of the applied frequency. The phase angle measures the time lag between the applied field and the subsequent particle motion [44]. Particle motion is adjusted by periodical polarization of the electrical double layer to form a dipole, as shown schematically in Fig. 3b. This phenomena is called the electrokinetic sonic amplitude or ESA effect. The first ESA device was invented by T. Oja et al. in early 1980 [60]. Figure 3a, b shows the ESA instrument and its relative parts, completed with SP-80 Probe (see Fig. 3b) which we have applied for direct characterization of high (77 mass%)-concentrated ZrO_2_ nanosuspensions. The magnitude and phase of the generated acoustic wave at the electrode-solution interface are related to the ζ-potential and polarity of the suspended particles.

The zeta-potential value is closely related to suspension stability and particle surface morphology [31, 61]. To calculate ζ-potential from the ESA data, several pieces of information are needed. For the suspending medium, the density, viscosity, and dielectric constant are required. For the particles, the density, volume fraction, and average size are necessary [59, 62]. The electroacoustic ESA technique offers many advantages over traditional microelectrophoresis methods: (1) measurements of electrophoretic mobility and ζ-potential at suspensions with the real solids loading can be made without sample dilution, (2) samples can be stirred during measurements to avoid settling, so automatic titrations in order to obtain zeta-potential vs. pH data are relatively easy to perform, (3) individual measurements are very rapid, allowing the user to track non-equilibrium behavior, (4) a pH-concentration profile for the adsorption of the dispersant onto low and concentrated suspensions of nanosize and ultrafine particle systems, in contrast with the multiple potentiometric titrations that are required by conventional techniques, could be quickly and semiautomatically obtained, and (5) the biggest advantage of ESA measurement, compared with the other techniques, such as laser light scattering, is the capability to characterize interacting concentrated dispersed systems without dilution. However, there are a few limitations which include (i) The precision of the ESA (ESA-8000, Matec) method depends on the applied frequency (MHz). (ii) The ESA signal for concentrated suspensions with volume fraction of above 20 vol.% is slightly lower at frequency of 1 MHz. (iii) Significant sources of experimental error may include the calibration procedure of the sample, such as the increased viscosity of the suspensions at higher solids loading, especially near the IEP (isoelectric point). These high viscosities can lead to poor mixing properties that result in sample heterogeneity during titrations. Therefore, it is advisable to determine the viscosity behavior of applied suspensions prior to ESA measurements [32, 35, 52].

In the present work, calibration and size correction were not performed, so the zeta-potential values reported are of a relative nature. In the experimental setup using ESA technique, two different sets of concentrated ZrO_2_ nanosuspensions were prepared, as follow:

The first set of suspensions was prepared at a low 10 mass% solids loading (*ϕ*~2 vol.%, dilute system) in distilled water with and without the addition of an appropriate amount of dispersant (Dolapix CE64) and measured directly (in Teflon vessel in the SSP-1 sample cell), see Fig. 3a.

The second set of suspensions was prepared at a high 77 mass% (*ϕ*~35.8 vol.%) solids loading in distilled water, containing different amounts of dispersant, which were progressively prepared in the same way for the rheological measurements. To achieve a better dispersing, the samples were mixed by magnetic stirrer for 30 min and sonicated for 3 min using an ultrasonic probe. For the first set of experiment, freshly prepared samples of low (10 mass %) concentrated suspensions were placed between two electrodes by partially immersing the electroacoustic (SP-80 probe) device into the liquid dispersion inside of the sample holder (Teflon vesel, SP-80 Cell) of the ESA-8000 device [see Fig. 3a], and allowed to equilibrate at 23 °C before ESA measurements [12].

For the second set of ESA measurements, meant to characterize the electrokinetic characteristics of high (77 mass%)-concentrated test nanosuspensions and to obtain better ESA signals, first, the ESA (SP-80) probe was aligned vertically. A small amount of suspension (20 ml) was then poured directly on the surface of ESA electrode that was large enough to accommodate ESA measurements at the same time, see Fig. 3b. This study indicates that this alignment helps to settle a very small volume of highly concentrated nanosuspensions on the ESA electrode and it minimizes challenging experimental errors during measurements. By this alignment charged, particles in highly concentrated suspensions could be settled on the surface of the ESA electrode in the same direction of the applied electric field and gravity. This helps to obtain better ESA signals. The reported values of the electrokinetic characteristics (i.e., ESA signal, ζ-potential and pH) were determined as an average of 5 measurements made under constant conditions for each suspension. To determine the ζ-potential as a function of pH for the two sets of prepared low- and high-concentrated ZrO_2_ nanosuspensions with and without the addition of dispersant Dolapix CE64, automatic titration was performed. Merck brand 0.1 N HCl and 0.1 N NaOH solutions were applied to adjust pH to the desired values from 3 up to 12, respectively. Experimental data for low concentrated (2 vol.%) nanosuspensions were recorded in steps of about 0.1 pH-units. For high (35.8 vol.%)-concentrated nanosuspensions, to prevent aggregation, insure pH stability, and determine the concentrations at which surface coverage is complete, pH was qualitatively determined just before and just after each ESA measurements. More detailed information on this method can be found in the literature [63].

#### Visualization of Dispersant-Coated Nanoparticles

**Fig. 4:**
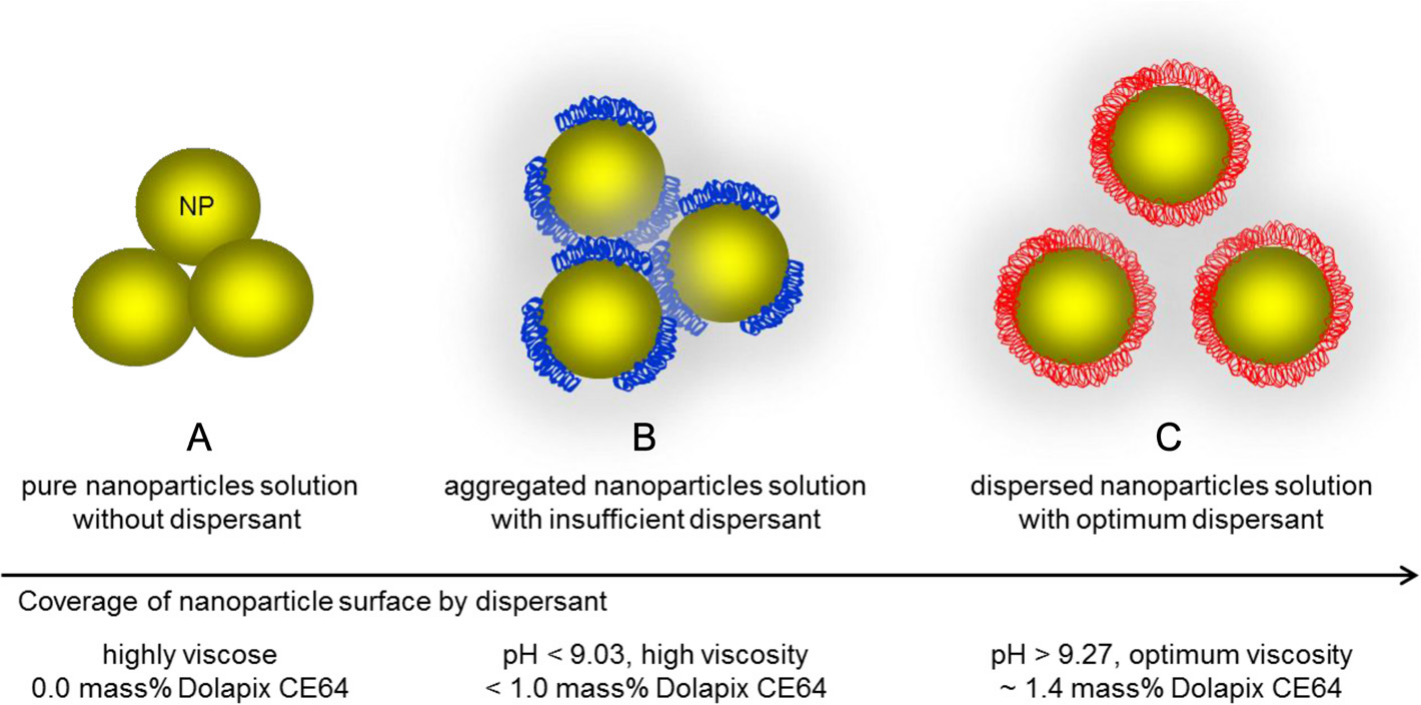
**a**–**c** Schematic nanoscale drawing of the dependence of dispersion stability on the dispersant concentration in high-concentrated ZrO_2_ nanosuspensions

**Fig. 5:**
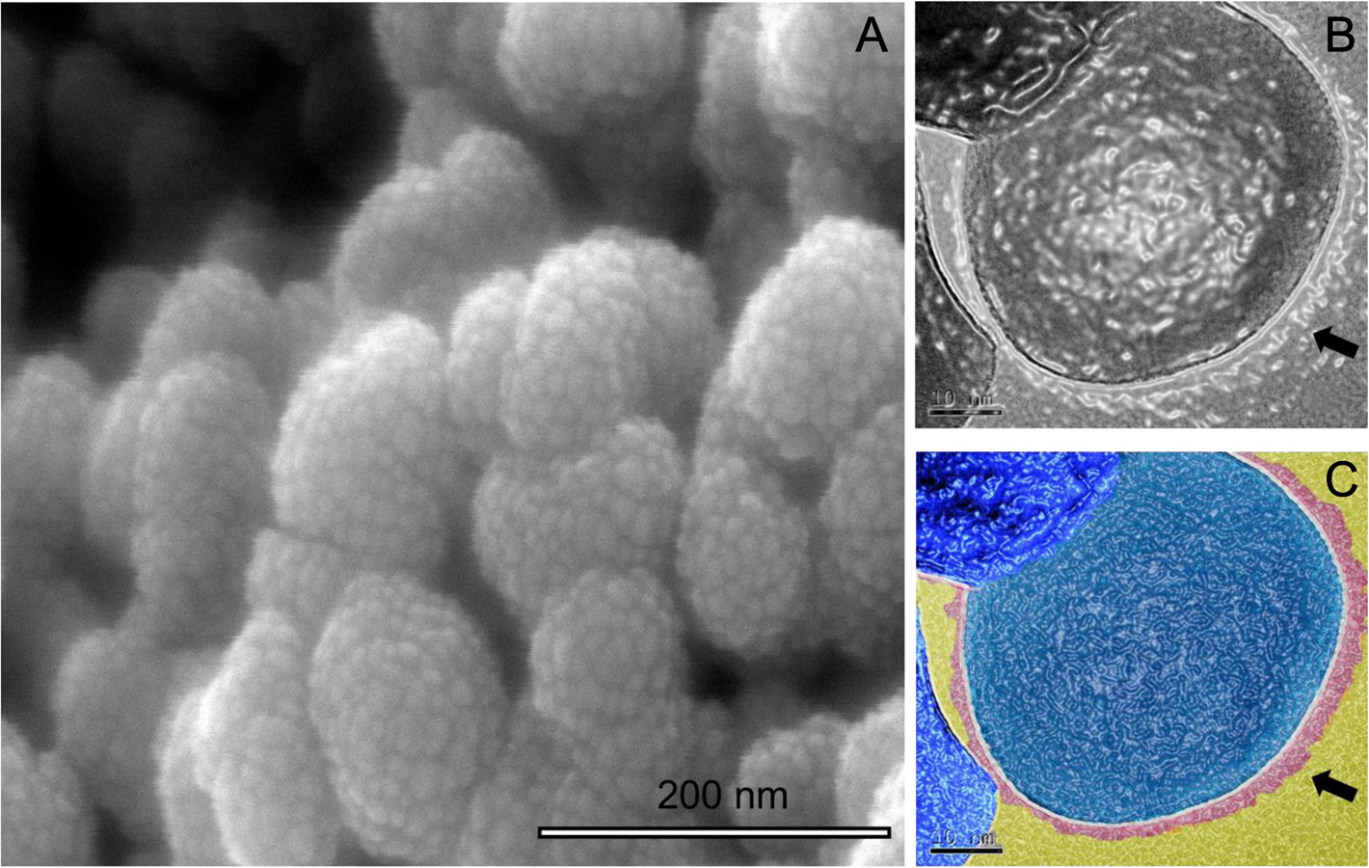
**a**–**c** A SEM image of ZrO_2_ nanoparticles coated/dispersed by dispersant Dolapix CE64, under the addition of optimum amount 1.4 mass% of dispersant (**a**), and colorized HR-TEM images (**b**, **c**) of coated ZrO_2_ nanoparticle by a very thin (1–6 nm) shell of Dolapix CE64. Indexes show the overall thickness of very thin (1–6 nm) shell of dispersant coated on the surface of nanoparticle. Two different parts of particle are distinguished by apparent color: The *dark* (image **b**) and *blue* (image **c**) parts represent the original particle. The *light* or *transparent* (image **b**) and *pink* (image **c**) one on the edge represent polyelectrolyte coating. Shell polyelectrolyte indicated by *arrows*

As a supplementary step, we have proposed a hypothesis that the addition of different amounts of dispersant influences both the stability and the electrokinetic and rheological properties of concentrated ZrO_2_ nanosuspensions. A schematic nanoscale model using ZrO_2_ nanoparticles as host particles and dispersant Dolapix CE64 as the coating polymer is shown in Fig. 4 (a, b, c). This hypothesis implies a dispersant effect in the formation of a fully stabilized suspension with the relative lower viscosity at pH >9.2 (see Fig. 4c) suitable for slip casting applications. To verify our hypothesis practically, scanning electron (SEM, Hitachi) and high-resolution transmission electron (TEM, JEOL JEM-2010F) microscopes were used at accelerating voltage of between 15–200 kV for observation of dispersant coating quality on the surface structure of the individual nanoparticles. SEM was used for visualization of dispersion quality of a concentrated 77 mass% ZrO_2_ nanosuspension which was dispersed with optimum amount of 1.4-mass% dispersant, see Fig. 5a. SEM sample was prepared by the suspension drop method with the use of SEM conductive carbon tape (Electron Microscopy Sciences, PA, USA): A drop of freshly ball-milled suspension was taken and dripped manually onto the carbon tape and dried for 24 h in air at room temperature. HR-TEM was used for surface visualization of individual nanoparticles before and after the addition of optimum amount of 1.4-mass% dispersant to a 2 vol.% as-received (bare) ZrO_2_ nanosuspension.

Two sets of TEM samples were prepared as follows: (i) A very diluted drop (0.002 vol.%) solution of 2.0 vol.% bare ZrO_2_ nanosuspension was deposited onto the carbon grid (Electron Microscopy Sciences, PA, USA) and dried in air before observation. (ii) A very diluted drop (0.002 vol.%) of dispersed (2.0 vol.% ZrO_2_−1.4 mass% Dolapix CE64) nanosuspension has been taken and was dripped manually onto the carbon grid (Electron Microscopy Sciences, PA, USA). The TEM grids were examined with an energy of 200 keV and magnifications of 10–300,000 times. False colors (based on colorization effect) were used to distinguish the interface between nanoparticle and potential coating dispersant layers. To false color TEM images (see Fig. 5b, c), a distinction between dispersant coating and the background was determined by a sharp difference in contrast, and a distinction between the nanoparticle (dark color in Fig. 5b and blue color in Fig. 5c) and the dispersant (pink color in Fig. 5c) coating was made by using the Fresnel fringes [64, 65] to identify thin layers of dispersant Dolapix CE64.

## Results and Discussion

### Effect of Dispersant Concentration on Rheology

**Fig. 6:**
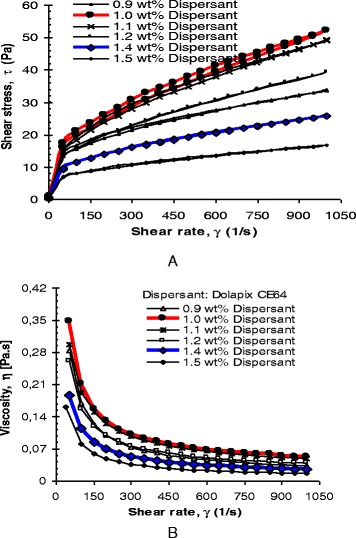
**a**, **b** The effect of dispersant (Dolapix CE64) concentration on the rheological behavior of concentrated (*ϕ*, ~77 mass%) ZrO_2_ nanosuspensions, as a function of shear rate, in the range of <0.03–1000 (s^−1^) >; **a** the flow curves, *τ*−*γ*; and **b** viscosity, *η* (Pa.s)−γ (s^−1^)

Figure 6a, b shows the rheological flow curves of (a) *τ* vs. *γ* and (b) *η* vs. *γ*, respectively, as a function of dispersant concentration for ZrO_2_ nanosuspensions with constant maximum solids loading of 77 mass %. All suspensions exhibit shear-thinning behavior with a reduction in viscosity at increasing shear rate, see Fig. 6(b). For suspension with 1.0-mass% dispersant, its *τ*–*γ* curve (Fig. 6a, denoted by red loop line) shows a typical flow shape that is observed in a strongly flocculated suspension: A high shear-thinning behavior with a high yield stress (typically at *γ* = 50 s^−1^), an irregular up-line, as well as with a large hysteresis loop of thixotropy. These characteristics indicate a poor dispersion of the suspension. This means that in spite of the insufficient amount of an electrosteric dispersant, bridging flocculation occurs. This is in agreement with our proposed model, see Fig. 4b. From Fig. 6b, it can be seen that viscosity is higher for suspension with 1.0-mass% dispersant (the curve denoted by red line), which is due to the insufficient amount of dispersant. More dispersant will be required to adsorb onto the surface of the particles to maintain a stable suspension. As the dispersant addition increases, the yield value decreases and the thixotropic loop diminishes. The *η*–*γ* depiction shows that shear-thinning diminishes as the amount of dispersant increases up to 1.4 mass% of Dolapix CE64 (Fig. 6b, denoted by blue line), as the optimum amount of dispersant concentration. This means that with the addition of optimum amount of an electrosteric dispersant, deflocculation occurs in a dispersed suspension. This is in agreement with our proposed hypothesis model, see Fig. 4c.

### Effects of Dispersant Concentration on Electrokinetic Properties

To determine the effect of dispersant concentration on electrokinetic properties (ESA signal, ζ-potential and pH) of different (2 and 35.8 vol.%) concentrated ZrO_2_ nanosuspensions, ESA measurements were carried out in two different concentrated nanosuspensions.

### Characterization of Low (2 vol.%)-Concentrated Suspensions

**Fig. 7:**
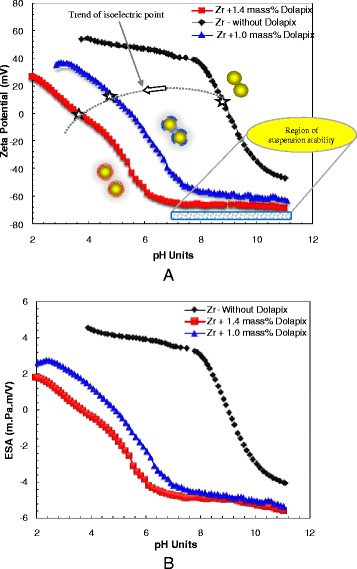
**a**, **b** ζ-potential (**a**) and ESA signal (m.Pa.mV^−1^) (**b**)–dependence on pH for ZrO_2_ nanosuspensions at a particle volume of 2 %, with and without the addition of optimum 1.4 mass% (red curve) and insufficient 1.0 mass% (blue curve) amounts of dispersant. Symbol *white star* shows isoelectric point

The electrokinetic properties (pH, zeta-potential, and ESA signal) of low-solids loading 2 vol.% ZrO_2_ nanosuspensions without and with addition of insufficient 1.0 mass% and optimum 1.4 mass% amounts (which were previously found on the basis of the viscosity measurement) of Dolapix CE64 were characterized. The results are shown in Fig. 7a, b and Table 2. The addition of dispersant has a strong effect on shifting of the pH at isoelectric point (pH_i.e.p_) from 8.96 for suspension without Dolapix to lower pH values of 4.83 and 3.61 for suspensions with insufficient (1.0 mass%) and optimum (1.4 mass%) amounts of Dolapix. The same influence is found to elevate magnitudes of ESA and zeta (ζ)-potential values. The graphs in Fig. 7a, b also show the pH dependence of zeta-potential and ESA in the pH range of 2–11 for low (2 vol.%) concentrated ZrO_2_ nanosuspensions with and without Dolapix CE64. Figure 7a shows the values of zeta-potential as a function of pH that were obtained from an electroacoustic titration of a 2-vol.% suspension of ZrO_2_ nanoparticles. Similar results were obtained for the ESA value which is proportional to ζ-potential [12, 32, 36, 44, 52], as shown in Fig. 7b. The upper curves in Fig. 7a, b show the pH dependence of ζ-potential and ESA in the pH range of 4–11 for ZrO_2_ suspension without dispersant (shown by black color). The lower curves in Fig. 7a, b show the ζ-potential and ESA values as a function of pH by the addition of insufficient (1.0 mass%, shown by blue color) and optimum (1.4 mass%, shown by red color) amounts of Dolapix CE64 to 2 vol.% ZrO_2_ nanosuspension. An agreement in graphical trend between the results from ζ-potential and ESA values as a function of pH was observed. A shift of the pH_iep_ to smaller pH values (shown by dotted line with gray color) was noted with an increase in the amount of dispersant up to its optimum content.

**Table 2:**
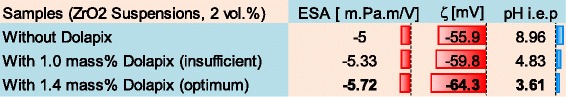
Electrokinetic characteristics of low (2 vol. %) concentrated ZrO_2_ nanosuspensions based on ESA, zeta-potential, and pH of I.E.P containing of different amounts of Dolapix CE64

The large plateau in the pH range of around 7.0–11 at optimum (1.4 mass%) dispersant concentration indicates a region of stability that is not strongly sensitive to pH. Further, the adsorption of the optimum amount of dispersant increased the magnitude of the negative charge of the ζ-potential (see Fig. 7a) and ESA (see Fig. 7b) values from their initial positive values. This increase in the negative (ζ) and ESA values is expected, because Dolapix CE64 is an anionic polyelectrolyte (with a pH of 9, reported by manufacturer) at all pH values in the range of 4–11 [32]. As a result, the surface charge of particles will change from a positive to a negative value at inherent pH of about 9 to 11. However, in this study, we indicate that the level of change in ESA value is small, mainly because the ESA 8000-probe was applied at a constant frequency of 1 MHz. At the same time, it is expected that at higher frequencies (1 < MHz <10), the ESA value will significantly be bigger [15].

It can also be inferred from the results, which by shifting the isoelectric point (pH_iep_) of pure powder suspension to a lower value by the addition of an optimum (1.4 mass%) amount of dispersant, dispersing stability increases. The shift in the isoelectric point is due to the fact that the negatively charged carboxylic groups dissociated from the dispersant are attached on the positively charged nanoparticle surface, based on the electrosteric stabilization effect, respectively [57].

In addition, it seems that the changes of electrokinetic characteristics (ζ-potentials, ESA values, and pH_iep_) of applied nanosuspensions are caused by the increase of the charging density of ZrO_2_ nanoparticles. This increase, in turn, is caused by the adsorption of negatively charged anion groups of dispersant on the surface of particles.

### Characterization of High (35.8 vol.%)-Concentrated Suspensions

In the second part of the ESA experiment, the electrokinetic (ESA, ζ-potential, and pH) characteristics of high (35.8 vol.%, ~77 mass%)-concentrated ZrO_2_ nanosuspensions with different amounts of dispersant in the range of 0.9–1.5 mass% were determined. The results are shown in Table 3. Table 3 shows the relationship between dispersant concentrations, ESA, zeta (ζ)-potential, and instant pH values of concentrated ZrO_2_ nanosuspensions. It can be seen that the magnitude of ESA, ζ-potential, and pH values increases as the dispersant content increases up to its optimum amount (1.4 mass%, sample TZ-1.4). The ESA, ζ-potential, and pH values attain their optimum limits at −12.30 (m.Pa.mV^−1^) and −8 (mV) and 9.27, as the value of dispersant approaches its optimum amount of 1.4 mass%. But, suspension (Table 3, sample TZ-1.0) with insufficient (1.0 mass%) amount of dispersant has smaller ESA, ζ-potential, and pH values of −10.984 (m.Pa.mV^−1^), 3.3 and 9.03.

**Table 3:**
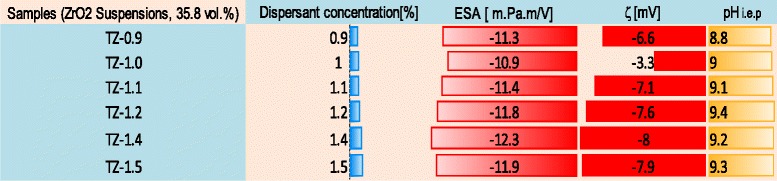
Electrokinetic characteristics of high-concentrated 35.8 vol.% (~77 mass%) ZrO_2_ nanosuspensions with different amounts of Dolapix CE64, measured by ESA

**Fig. 8:**
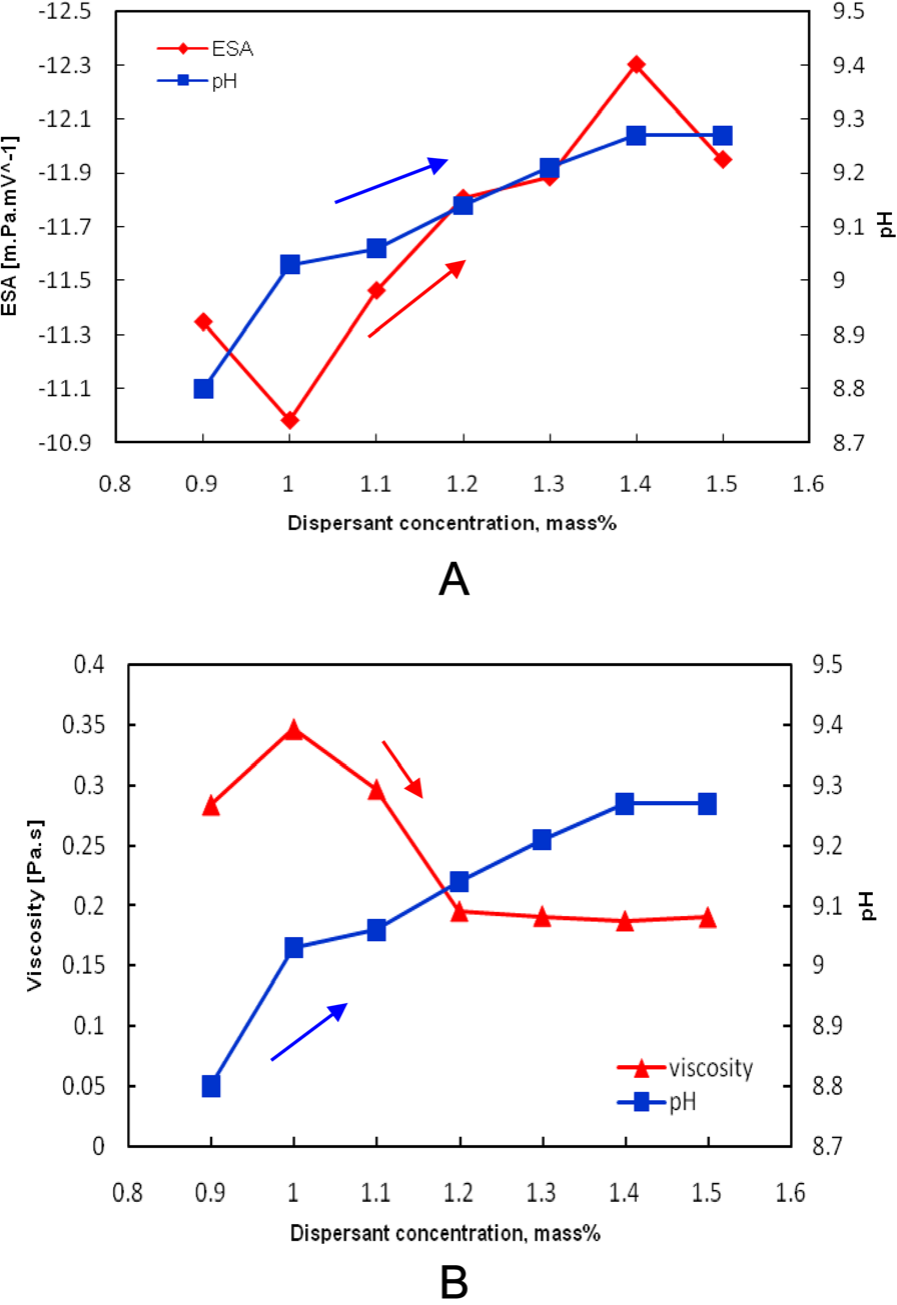
**a**, **b** Relationship between **a** ESA and pH magnitudes and **b** viscosity and pH magnitudes at the shear rate of 50 s^−1^ of high-concentrated ZrO_2_ nanosuspensions (with constant 77-mass% solids loading) as a function of dispersant concentration

**Fig. 9:**
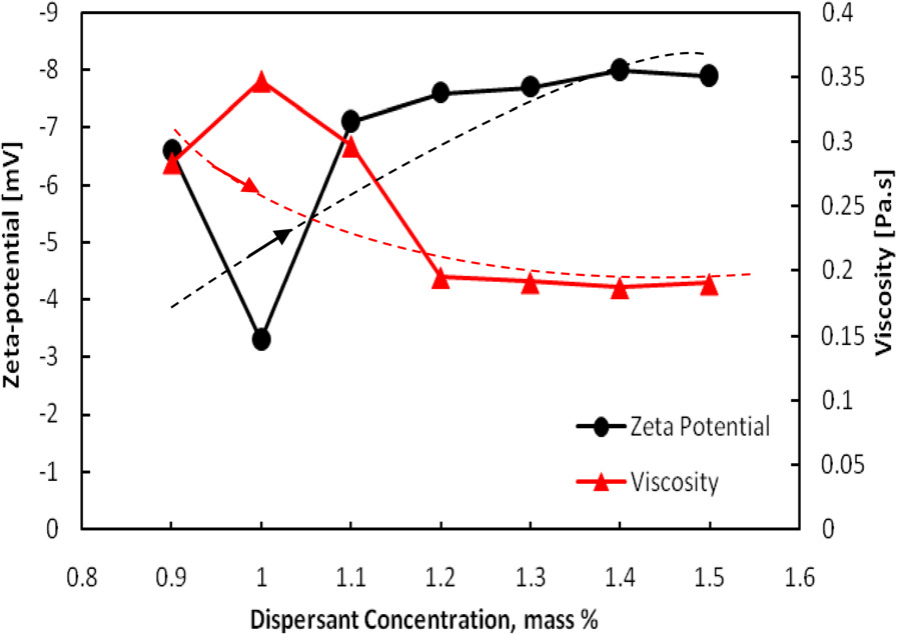
Relationship between ζ-potential and viscosity magnitudes at the shear rate of 50 s^−1^ of high-concentrated ZrO_2_ nanosuspensions (with constant 77-mass% solids loading) as a function of dispersant concentration

A comparative investigation conducted to determine the relationship between ESA, pH, and suspension viscosity values (at the shear rate of 50 s^−1^) of concentrated 77 mass% ZrO_2_ nanosuspension as a function of dispersant concentration is shown in Fig. 8a, b. The comparison of the results obtained from Table 3, and Fig. 8a, b confirms that the amount of 1.4 mass% Dolapix CE64 is the optimum amount of anionic polyelectrolyte that can be adsorbed onto the surface of ZrO_2_ nanoparticles, leading to stabilize a concentrated ZrO_2_ nanosuspension at the inherent pH >9.2. This result is also confirmed by comparing the zeta-potential and suspension viscosity as a function of dispersant concentration in the range of 0.9–1.5 mass% on concentrated 77 mass% ZrO_2_ nanosuspensions, see Fig. 9. It is evident that viscosity (*η*) presents a minimum (~0.18 Pa.s) at shear rate of 50 s^−1^ and ζ-potential value maximum (−8 mV) at 1.4 mass% of dispersant corresponding to the optimum powder dispersion. In addition, the results from Figs. 8 and 9, and Table 3 indicate that at higher concentrations of dispersant (>1.4 mass%, above the optimum value), the viscosity increases slightly and the magnitude of ζ-potential value decreases, relatively. It can be concluded that the addition of optimum amount 1.4 mass% of Dolapix CE64 to a concentrated 77 mass% ZrO_2_ nanosuspension at pH 9.27 provided electrosteric dispersion. It is in good agreement with our proposed hypothesis and recently reported results from adsorption analysis [32, 47]. Further, as it can be seen in Fig. 9, we also consider that both the zeta-potential and viscosity are relatively constant between dispersant concentrations of 1.2 and 1.5 mass%, with an optimum value for both parameters occurring at a concentration of 1.4 mass%.

### Microscopic Imaging of Dispersant Coating Nanoparticle

Figure 5a shows an SEM image of ZrO_2_ nanoparticles coated/dispersed by an optimum amount of 1.4 mass% of dispersant Dolapix CE64. Figure 5b shows HR-TEM image of an individual ZrO_2_ nanoparticle surrounded by a bright observation (shown by pink color) confirming dispersant coating of the ZrO_2_ nanoparticle surface under the addition of optimum amount 1.4 mass% of dispersant. In Fig. 5b, two different parts of the nanoparticle are distinguished by apparent colors: The blue part represents the original particle, and the pink light or transparent one on the edge represents dispersant coating covered nanoparticle surface. The overall thickness of the shell polymer coating (shown by pink color) on the nanoparticle surface is very thin (a few nanometers or a few atomic layers) and indicated by arrows. In Fig. 1b, the lattices of bare ZrO_2_ nanoparticle are visible. But in Fig. 5b, the lattices of ZrO_2_ nanoparticle are not visible due to dispersant coating. From SEM and HR-TEM images presented in Fig. 5a, b, it can be inferred that coating of the ZrO_2_ nanoparticles based on electrosteric stabilization appears to be possible. This is in agreement with our proposed hypothesis and a recent work reported by K. Verhiest et al. [46]. However, as it can be seen from Fig. 5b, it is very complex and difficult to observe the image of a polymer shell homogenously coating the nanoparticle surface, due to overlap in three dimensions of particle structures [66]. Therefore, it is necessary to indicate that obtaining a high-resolution TEM image of an individual nanoparticle coated homogenously by polymer is a very tedious and time consuming process [67].

## Conclusions

The effect of anionic polyelectrolyte Dolapix CE64 concentration on the dispersion stability of two concentrated ZrO_2_ nanosuspensions (10 and 77 mass%) was studied in terms of direct characterization of rheological and electrokinetic properties by using a Haake rheometer and electroacoustic sonic amplitude ESA technique. The following conclusions were evidenced:

- In both low (10 mass%)- and high (77 mass%)-concentrated colloidal batches, stable ZrO_2_ nanosuspensions were obtained by adding 1.4 mass% of anionic polyelectrolyte.
- Substantial agreement is observed between ζ-potential, ESA signal, viscosity, and the relative pH dependency for concentrated ZrO_2_ nanosuspensions with different amounts of polyelectrolyte.
- The results obtained from the relationship between the magnitudes of ζ-potential, ESA signal, pH, and viscosity indicate that 1.4 mass% Dolapix CE64 is the optimum amount of dispersant that can be absorbed onto the surface of ZrO_2_ nanoparticles leading to stabilize both low (10 mass%)- and high (77 mass%)-concentrated ZrO_2_ nanosuspensions at the inherent pH >9.2 with strong electrosteric dispersion effect. This is in good agreement with our proposed hypothesis of colloidal model.
- Supplementary SEM and HR-TEM techniques accompanied with colorization of polymer coated nanoparticles provide a qualitative solution to evaluate the effect of dispersant concentration.

The results of this work point out that electroacoustic ESA technique can be useful when applied in combination with other traditional techniques such as rheometry for direct probing stability and characterization of dispersion properties of low- and high-concentrated nanoparticle suspensions close to the processing conditions. Apart from this, the rapidity of the electroacoustic ESA technique allows the prospect of real-time process control in production line. More work is also required to correlate dilute and concentrated systems and to determine the origin of certain material-dependent variations.

It is expected that using electroacoustic ESA technique would be useful for direct probing of electrokinetic properties not only of inorganic materials but also nanomaterials such as carbon nanotubes. It is important to consider that the accuracy of ESA technique will depend on the applied frequency, characteristics of different materials, and their specific rheological and electrokinetic properties. Therefore, the potential use of ESA technique for direct characterization of various concentrated colloidal systems without dilution is challenging and will be further explored by improving the accuracy of the parameters and devices.

## Abbreviations

(ζ): zeta-potential
Dolapix CE64: a commercial name of an anionic polyelectrolyte dispersant
ESA: electrokinetic sonic amplitude
HR-TEM: high-resolution transmission electron Mmicroscope
IEP: isoelectric point
SEM: scanning electron microscope
